# Hospital Abuse and Medical Discontent

**Published:** 1906-11-10

**Authors:** 


### The Hospital

A Journal of

The Medical Sciences and Hospital Administration.

Vol. XLI.—No. 1,051. SATURDAY, NOVEMBER 10, 1906.

### Hospital Abuse and Medical Discontent.

General practitioners, finding it more and more difficult to make a practice, and, when they have purchased one, to maintain the receipts at a sum sufficient to pay expenses and to leave some margin for maintenance, have become increasingly discontented in recent years. They, or some of them who purport to represent the general body, have taken action, in various ways, as members of the British Medical Association, some of which were set forth in The Hospital for October 20, 1906, pages 59 *et seq.* The *Daily Telegraph* and other newspapers have recently published considerable correspondence on the subject. There can be no doubt that the general practitioners are justified in exerting themselves to remove the causes which have created, and do in fact underlie, the present evils. These causes are capable of precise definition, and in our judgment are readily remediable, providing the general practitioners will act together, and on a definite plan, which must ensure the unfailing application of the needed remedy, at the source of origin.

First let us point out that no practical results can be expected, from a reiteration of the old and oft-repeated remedies, which have failed to win the support of those responsible, and which could never prove effective in practice. Many of these matters are fully dealt with in the article just referred to. But the condition depends essentially upon (1) the excessive free medical relief at the hospitals; (2) the visiting medical staffs of hospitals, whose inaction gives encauragement to, and prevents a drastic cutting down of, the number of free patients; and (3) the consequent and increasing feeling on the part of the public, that everybody, when sick, is entitled, if they choose, to claim free hospital relief.

The abuse of medical relief is great indeed, for although the population of the administrative county of London only increased 6 per cent. in the ten years ending 1903, the percentage of increase in the hospital population amounted to 23.21 per cent. When the Outer Ring and the hospitals it contains are included—*i.e.* every parish, the whole of which is within fifteen miles of Charing Cross, or any portion of which is within twelve miles—the evidence of excessive free medical relief by the hospitals does not diminish but increases in force. The figures show that the population of the administrative county, including the outer ring—that is, the whole area of Greater London, covering nearly 700 square miles—increased in the ten years ending 1903, by 14 per cent., whilst the number of patients treated at all the hospitals, situated within the same area, increased by 25.5 per cent. In fact, then, the voluntary hospitals in Greater London have actually undertaken 81 per cent. more work, in the decade, than the increase in the population warrants. We do not propose to labour these figures, but they must convince any searcher after truth, that the whole system of free medical relief in Greater London, so far as the voluntary hospitals are concerned, requires reconsideration and reconstruction.

Dealing next with the second head, it is the fact, that in practice, with the abolition of the ticket system, the admission or rejection of patients, at the voluntary hospitals, has passed into the hands of the visiting medical staff. They have, at present, practically unlimited power to admit or reject patients, and it is a common practice for cases to be sent up by general practitioners in the country, to a member of the honorary staff, though, in a business sense, the patient has no proper claim to the benefits of a London hospital, at all. These country cases are held at some hospitals to have a paramount claim and in fact often obtain admission as in-patients, to the exclusion of the local out-patient cases who may have been set aside on the same day as eligible for admission. It has been held to be essential to encourage persons to apply to the hospitals, where there is a medical school attached, in order to secure the widest area of selection for the clinical cases essential to successful teaching. This argument has, we think, admitedly lost some of its force of late, but it is still held by some members of the visiting staffs. We have never admitted its justification, knowing the paramount and just claims, that the general practitioners have upon the whole-hearted co-operation of every honorary medical officer of a voluntary hospital. The public have learnt to appreciate the enormous improvements and developments in the modern hospital, and now hold, we think rightly, that when a patient is really ill the best place for him, after all, is a hospital. This would be all very well, if every hospital had, as it ought to have, a pay wing, but in existing circumstances it leads to an abuse of charity, which does material injury to the general practitioner.

What, then, is the economical side of the present medical discontent? The consultants, who collectively represent the medical staffs of the hospitals, depend largely, for their income as consultants, upon the patients sent to them by the general practitioners. The position and prospects of the consultant were probably never weaker than they are to-day. This fact is due to the improvements which have taken place in medical education. Many general practitioners have had an equally good training, and possess equally high degrees with the consultants. Graduate instruction now enables every general practitioner, who has the ability and brains, to place himself, so far as mere knowledge is concerned, upon a level with the consultant. Indeed, every hospital, with a medical school, would be well advised to organise a system of free graduate instruction for qualified practitioners, who have been students at such hospital, where they should always be welcomed as students. On this plan the hospitals would gain the whole-hearted support of a body of influential men, scattered all over the country, who could collectively increase the revenue of such an institution greatly. For economic reasons, too, as we have shown, the consultants, who collectively constitute the medical staffs of the hospitals, should, as an ordinary matter of business, act in co-operation with the general practitioner, and do what they can without delay effectually to reduce the present excessive free hospital relief to a minimum.

The remedy and its application are thus ready to hand. The committees, though very willing to make the voluntary hospitals efficient, as matters stand at present, are largely in the hands of the visiting medical staffs. The general practitioners, alone, have it in their power to bring sufficient pressure to bear upon the visiting medical staffs, and so to ensure the removal of the present hospital abuses, in regard to free medical relief. It is to the interest of the consultant, that the visiting staffs should curtail this free relief, not only as a matter of business, but as a matter of duty to the committees of management, who have to raise the money for the hospitals, from the public. By such action, and by such action alone, can hospital abuse be effectually handled. Were this plan once adopted, the moneys available for the support of the voluntary hospitals should be found adequate. They should prove ample to defray the cost of providing the maximum of free medical relief, to those citizens, only, whose medical needs and social position justify their acceptance of this gratuitous service.

